# Validation and modification of staging Systems for Poorly Differentiated Pancreatic Neuroendocrine Carcinoma

**DOI:** 10.1186/s12885-020-6634-9

**Published:** 2020-03-06

**Authors:** Haihong Wang, Zhenyu Lin, Guiling Li, Dejun Zhang, Dandan Yu, Qili Lin, Jing Wang, Ye Zhao, Guoliang Pi, Tao Zhang

**Affiliations:** 1 Cancer Center, Union Hospital, Tongji Medical College, Huazhong University of Science and Technology, Wuhan, 430022 China; 2 School of Business and Administration, Jiangxi University of Finance and Economics, Nanchang, China; 3 Department of Radiation Oncology, Hubei Cancer Hospital, Tongji Medical College, Huazhong University of Science and Technology, Wuhan, 430079 China

**Keywords:** Neoplasm staging, SEER program, Pancreatic neuroendocrine carcinoma, Poorly differentiated

## Abstract

**Background:**

The American Joint Committee on Cancer (AJCC) and the European Neuroendocrine Tumor Society (ENETS) staging classifications are two broadly used systems for pancreatic neuroendocrine tumors. This study aims to identify the most accurate and useful tumor–node–metastasis (TNM) staging system for poorly differentiated pancreatic neuroendocrine carcinomas (pNECs).

**Methods:**

An analysis was performed to evaluate the application of the ENETS, 7th edition (7th) AJCC and 8th edition (8th) AJCC staging classifications using the Surveillance, Epidemiology, and End Results (SEER) registry (*N* = 568 patients), and a modified system based on the analysis of the 7th AJCC classification was proposed.

**Results:**

In multivariable analyses, only the 7th AJCC staging system allocated patients into four different risk groups, although there was no significant difference. We modified the staging classification by maintaining the T and M definitions of the 7th AJCC staging and adopting new staging definitions. An increased hazard ratio (HR) of death was also observed from class I to class IV for the modified 7th (m7th) staging system (compared with stage I disease; HR for stage II =1.23, 95% confidence interval (CI) = 0.73–2.06, *P* = 0.44; HR for stage III =2.20, 95% CI =1.06–4.56, *P* = 0.03; HR for stage IV =4.95, 95% CI =3.20–7.65, *P* < 0.001). The concordance index (C-index) was higher for local disease with the m7th AJCC staging system than with the 7th AJCC staging system.

**Conclusions:**

The m7th AJCC staging system for pNECs proposed in this study provides improvements and may be assessed for potential adoption in the next edition.

## Background

Pancreatic neuroendocrine tumor is a rare malignancy with great heterogeneity, which presented with variable biologic behavior ranging from benign or indolent to frankly malignant or aggressive [1]. Its incidence has been increasing sharply in recent years, partly due to the increased application of computed tomographic scans and endoscopic technologies [2, 3]. According to tumor morphology and markers of proliferation, neuroendocrine neoplasms of the pancreas (pNENs) are divided into well-differentiated pancreatic neuroendocrine tumors (pNETs) and poorly differentiated pancreatic neuroendocrine carcinomas (pNECs) [4]. It is reported that pNECs only account for about 15% of pNENs [5–7], and several studies have shown that the clinicopathological features, prognosis and gene expression between pNECs and pNETs are completely different [1, 8–11]. Thus, due to their rarity and heterogeneity, pNECs have not been well studied, and standard staging tools have been lacking. Therefore, a staging system that can accurately provide prognostic information and stratify patients by risk is urgently needed.

TNM staging, an accurate and simple instrument for prognosis assessment and patient management at diagnosis for physicians, is the most frequently used indicator of outcomes for malignancies. Currently, there are two major different TNM-based staging systems for pNENs in use. A TNM staging system specially for pNENs was first proposed in the year 2006 by the ENETS [12]. This staging system was validated to risk stratify patients and discriminate among prognostic groups for pNENs by several studies [2, 13–19]. However, the vast majority of participants used to inform the ENETS staging classification were diagnosed with pNETs. Thus, the ENETS staging system has been recommended by the National Comprehensive Cancer Network guidelines for pNETs. In 2010, the 7th AJCC staging system first staged pNENs and employed the same staging system as they used for exocrine pancreas malignancies [20–22]. The system was also validated in several independent series for pNENs [2, 13, 23]. More recently, the 8th AJCC staging system on pancreatic ductal adenocarcinoma, which was released in October, 2016, has been recommended to replace the old version (the 7th AJCC) [24]. The tumor definitions and derived stages of the ENETS, 7th AJCC and 8th AJCC staging systems differ greatly (Supplementary Table 1). Moreover, the two AJCC staging systems are the same as for the ductal adenocarcinoma and not meant for pNECs. Therefore, the question regarding the suitable staging system specially for pNECs remain unanswered.

The majority of the research in the literature consists of small studies focusing on a few single aspects of the disease or larger studies where only a few cases of pNECs are included in a larger pNEN-cohort. This study was performed to analyses the performance of the ENETS,7th and 8th AJCC staging classifications when a large series of pNECs were used, and test a new modified staging classification that would address the weaknesses associated with 7th staging classifications system.

## Methods

### Patients and data collection

The data used in our study were retrieved between 1973 and 2015 from the SEER database of the US National Cancer Institute. Primary site labels “C25.0-C25.4” and “C25.7-C25.9” were used. Eligible patients were those diagnosed with pathologically confirmed poorly differentiated or undifferentiated pNENs, who were identified using the following International Classification of Diseases for Oncology (3rd edition) codes: 8150/3, 8151/3, 8152/3, 8153/3, 8155/3, 8156/3, 8157/3, 8240/3, 8241/3, 8242/3, 8243/3, 8246/3, and 8249/3. Patient demographics (race, sex, age at diagnosis, year of diagnosis, survival months, SEER cause-specific death classification) were included. TNM information was retrieved based on the following codes: derived AJCC stage group (7th edition; 2010+), derived AJCC stage group(6th edition; 2004+), collaborative stage (CS) tumor size (2004+),CS extension (2004+), CS lymph nodes (2004+), CS metastases at dx (2004+), regional nodes examined (1988+), regional node positive (1988+), derived SEER summary stage 1977 (1995–2000),SEER summary stage 2000 (2001–2003),Summary stage 2000 (1998+),extent of disease (EOD) 10 - nodes (1988–2003),EOD 10 - extent (1988–2003),and EOD 10 - size (1988–2003). We excluded those who had: unknown follow-up information, unknown cancer stage at diagnosis and any other primary tumors. The patients with unknown extent of tumor or lymph node status were included in our study if they had distant metastases.

### Statistical analysis

Several different statistical methods were applied to compare different staging schemes. We used Kaplan–Meier method to estimate tumor-related death-free survival. Patients dying from causes other than their cancer were censored at their date of death. Multivariate analysis of each staging classification, controlling for race, sex, age and tumor location was performed using cox proportional hazards regression. HRs and 95% CIs were calculated. C-indices were calculated to evaluate the discriminatory powers of the two staging systems between the 7th AJCC and the m7th AJCC staging systems for pNECs. A C-index of 1 represents perfect discrimination, and a C-index of 0.5 means agreement by chance alone [25]. Analyses were performed using SPSS version 22.0 and R version 3.5.1. All results are from 2-sided hypothesis tests with the significance level set to 0.05.

## Results

### Patients characteristics

A total of 644 eligible patients with pathologically confirmed pNECs were identified from the SEER database. And 75 cases were excluded for unknown cancer stage at diagnosis, 1 for unknown follow-up information. In total, 568 patients were included in the study. Table 1 shows frequency distributions of selected characteristics for the full study cohort.

**Table 1:** Baseline Clinicopathologic Characteristics

Characteristic	*N* = 568	
	No.	%
Age, years		
< 60	231	40.7
≥ 60	337	59.3
Sex		
Male	335	59.0
Female	233	41.0
Race		
White	443	78.0
Black	67	11.8
Other	58	10.2
Year of diagnosis		
1988–1997	60	10.6
1998–2007	187	32.9
2008–2015	321	56.5
Location		
Head	236	41.5
Body and tail	179	31.5
Other*	153	27.0
ENETS stage		
I	7	1.2
II	34	6.0
IIA	15	2.6
IIB	19	3.4
III	113	19.9
IIIA	25	4.4
IIIB	88	15.5
IV	414	72.9
8th AJCC stage		
I	35	6.2
IA	8	1.4
IB	27	4.8
II	78	13.7
IIA	27	4.8
IIB	51	8.9
III	41	7.2
IV	414	72.9
7th AJCC stage		
I	33	5.8
IA	8	1.4
IB	25	4.4
II	102	18.0
IIA	23	4.1
IIB	79	13.9
III	19	3.3
IV	414	72.9
Modified 7th stage		
I	50	8.8
IA	9	1.6
IB	41	7.2
II	85	15.0
III	19	3.3
IV	414	72.9

*Other location included overlapping lesion of pancreas and other specified parts of pancreas

The median age at diagnosis was 63.0 years (mean 62.0 years). Male patients account for a slightly higher proportion than female patients (a male to female ratio of 1.4:1.0). More than three quarters of the patients were white. In addition, 41.5% of the patients had tumors located at the head of the pancreas. A total of 418 patients died of their cancer. The estimated median overall survival (OS) was 10.0 months.

### ENETS staging classification and survival

**Fig. 1:**
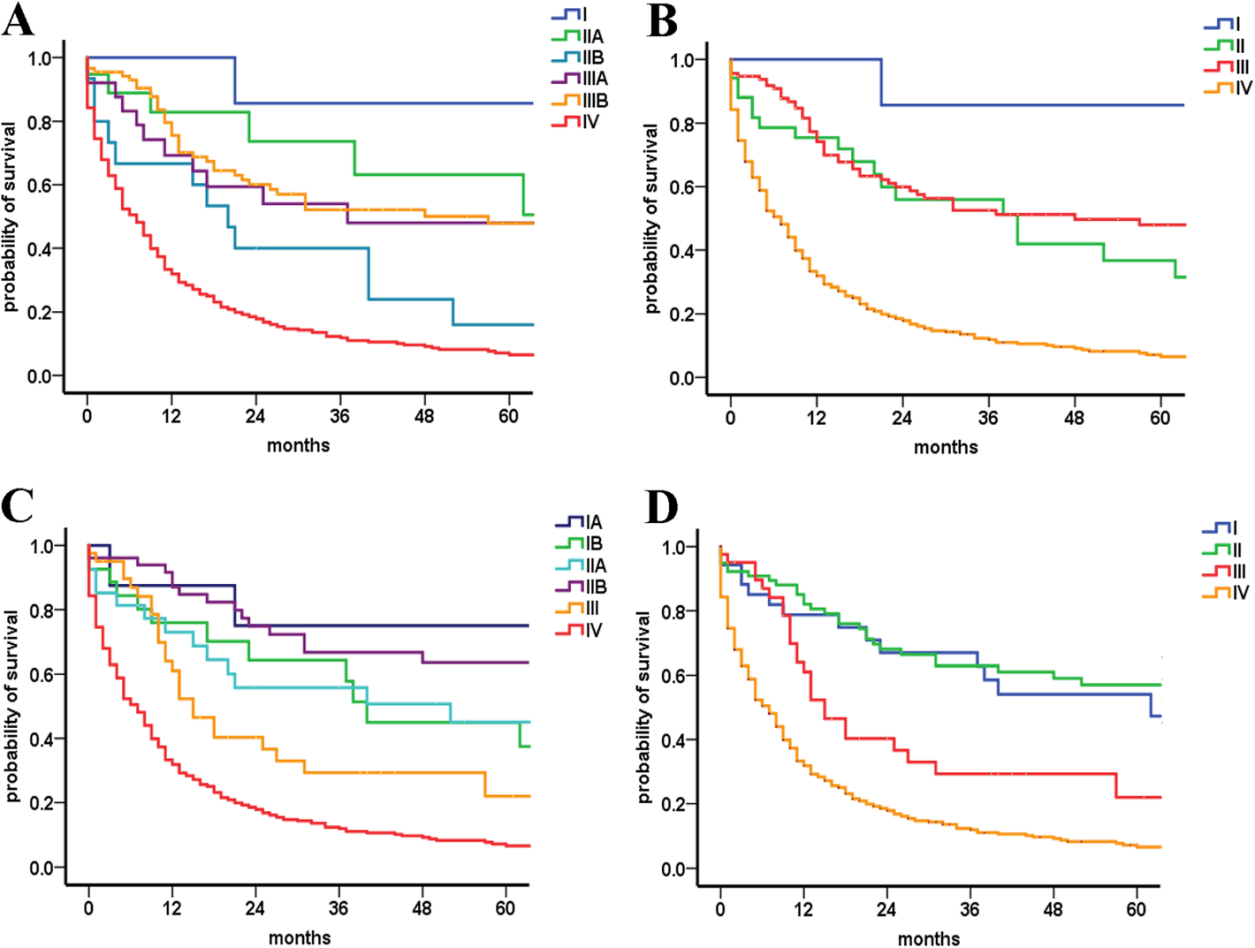
Kaplan-Meier curves of different staging classifications for patients from the SEER database. (**a** and **b**) European Neuroendocrine Tumor Society (ENETS) staging system; (**c** and **d**) 8th American Joint Committee on Cancer (AJCC) staging system

According to the ENETS staging classification, only 1.2% (7 of 568) of patients had stage I tumors and 6.0% (34 of 568) of patients had stage II tumors (Table 2). Overlap was noticed for the ENETS classification of stage II and III disease (Fig. 1a-b). In addition, median OS uniformly decreased from class I to II, was longer in class III, and decreased further in class IV. The median OS for stage I, II, III and IV were 90.0, 40.0, 48.0 and 7.0 months, respectively (Table 2). Compared with stage I disease, the HR of stage II was comparable to that of stage III (stage II and III HRs, 3.27 and 2.23, respectively) by multivariable analyses (Table 3).

**Table 2:** Univariate Analyses of tumor-related death among 568 patients using the ENETS, 8th AJCC, 7th AJCC and m7th AJCC staging systems including four tumor stages for pNECs

TNM staging systems	Median OS (months)	HR (95%CI)	*P*
ENETS			
I	90.0	1	
II	40.0	3.28 (0.77–13.98)	0.11
III	48.0	2.22 (0.54–9.13)	0.27
IV	7.0	8.92 (2.21–35.92)	0.002
8th AJCC			
I	62.0	1	
II	138.0	0.70 (0.38–1.26)	0.23
III	15.0	1.73 (0.93–3.21)	0.08
IV	7.0	3.70 (2.26–6.06)	< 0.001
7th AJCC			
I	90.0	1	
II	40.0	1.08 (0.60–1.93)	0.79
III	13.0	2.14 (0.98–4.67)	0.06
IV	7.0	4.36 (2.58–7.36)	< 0.001
m7th AJCC			
I	90	1	
II	31	1.24 (0.74–2.07)	0.41
III	13	2.31 (1.12–4.75)	0.02
IV	7	4.70 (3.05–7.24)	< 0.001

Abbreviations: *AJCC* American Joint Committee on Cancer; *ENETS* European Neuroendocrine Tumor Society; *m7th AJCC* modified 7th AJCC; *pNECs* poorly differentiated pancreatic neuroendocrine carcinomas; *OS* overall survival; *HR* hazard ratio; *CI* confidence interval

**Table 3:** Multivariate Analyses of Prognostic Factors of tumor-related death among 568 patients using the ENETS, 8th AJCC, 7th AJCC and m7th AJCC staging systems including four tumor stages for pNECs

Characteristic	ENETS		8th AJCC		7th AJCC		m7th AJCC	
	HR (95% CI)	*P*	HR (95% CI)	*P*	HR (95% CI)		HR (95% CI)	*P*
Age, years								
< 60	1		1		1		1	
≥ 60	1.63 (1.33–2.00)	< 0.001	1.64 (1.34–2.01)	< 0.001	1.66 (1.35–2.03)	< 0.001	1.66 (1.35–2.03)	< 0.001
Sex								
Female	1		1		1		1	
Male	1.16 (0.95–1.41)	0.15	1.16 (0.95–1.41)	0.15	1.14 (0.94–1.40)	0.18	1.14 (0.94–1.40)	0.18
Race								
Black	1		1		1		1	
White	0.65 (0.48–0.88)	0.01	0.66 (0.49–0.90)	0.01	0.67 (0.50–0.91)	0.01	0.67 (0.50–0.91)	0.01
Other	0.65 (0.43–0.99)	0.04	0.68 (0.45–1.04)	0.07	0.68 (0.45–1.03)	0.07	0.68 (0.45–1.03)	0.07
Location								
Body and tail	1		1		1		1	
Head	1.63 (1.29–2.08)	< 0.001	1.61 (1.27–2.05)	< 0.001	1.60 (1.26–2.03)	< 0.001	1.60 (1.26–2.04)	< 0.001
Others	1.67 (1.29–2.15)	< 0.001	1.68 (1.30–2.17)	< 0.001	1.67 (1.21–2.15)	< 0.001	1.67 (1.29–2.16)	< 0.001
Stage								
I	1		1		1		1	
II	3.27 (0.77–14.02)	0.11	0.74 (0.41–1.34)	0.32	1.08 (0.60–1.94)	0.80	1.23 (0.73–2.06)	0.44
III	2.23 (0.54–9.18)	0.27	1.81 (0.97–3.38)	0.06	2.06 (0.94–4.52)	0.07	2.20 (1.06–4.56)	0.03
IV	9.59 (2.38–38.71)	0.002	4.14 (2.52–6.79)	< 0.001	4.62 (2.73–7.83)	< 0.001	4.95 (3.20–7.65)	< 0.001

Abbreviations: *AJCC* American Joint Committee on Cancer; *ENETS* European Neuroendocrine Tumor Society; *m7th AJCC* modified 7th AJCC; *pNECs* poorly differentiated pancreatic neuroendocrine carcinomas; *HR* hazard ratio; *CI* confidence interval

### The 8th AJCC staging classification and survival

It is notable that overlap existed between the 8th AJCC classification of stage I and II disease (Fig. 1c-d). The median OS, in more detail, for stage I, II, III and IV were 62, 138, 15.0 and 7.0 months, respectively (Table 2). No statistical significance was observed for HR between stage I and stage II disease by multivariable analyses (stage I served as the reference; stage II HR, 0.74; *P* = 0.32; Table 3).

### The 7th AJCC staging classification and survival

**Fig. 2:**
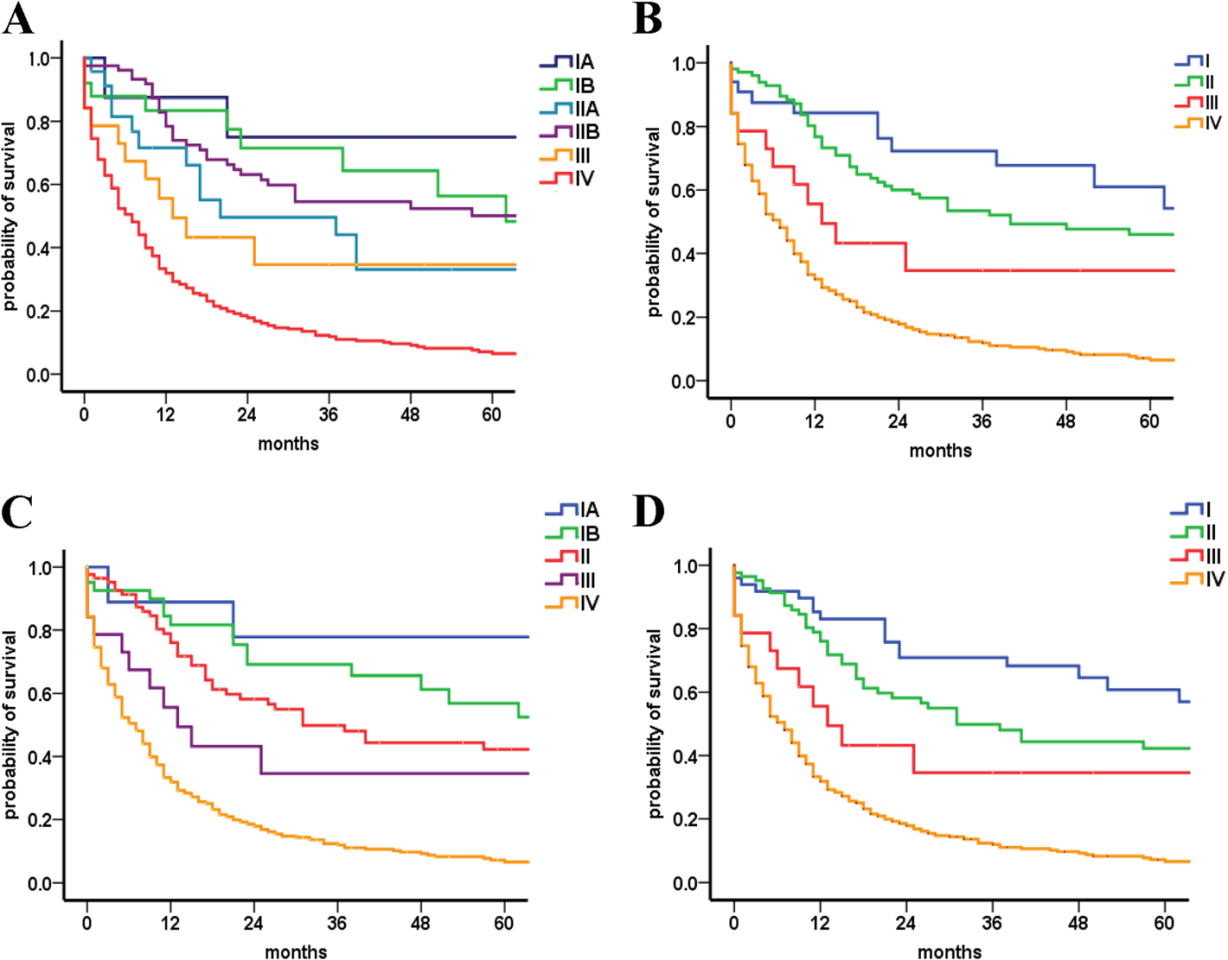
Kaplan-Meier curves of different staging classifications for patients from the SEER database. (a and b) 7th American Joint Committee on Cancer (AJCC) staging system; (**c** and **d**) modified 7th AJCC staging system

For the 7th AJCC staging system, the median OS uniformly decreased from class I to IV (Table 2), although overlap was also noticed for the 7th AJCC classification of stage I and II disease (Fig. 2a-b). Furthermore, the median OS of the patients with the same tumor stage varied widely between the different substages. Within stage II of the current AJCC staging system, the median OS for T3N0M0 and T1-3N1M0 were 20.0 and 78.0 months, respectively. The death risk of the patients increased from stage I to IV by multivariable analyses (stage I served as the reference; stage II HR of death = 1.08, 95% CI = 0.60 to 1.94, *P* = 0.80; stage III HR of death = 2.06, 95% CI = 0.94 to 4.52, *P* = 0.07; stage IV HR of death = 4.62, 95% CI = 2.73 to 7.83, *P* < 0.001; Table 3).

### Comparison of survival outcomes based on the current and modified 7th AJCC staging systems

Considering the shortcomings of the AJCC and ENETS systems cited previously, a m7th AJCC staging classification was proposed by maintaining the T and M definitions of the 7th AJCC staging system and adopting a new staging definition system. Stage IV is the same across all systems defined as disease with distant metastasis. The proportion of patients with stage I disease using the m7th AJCC staging system was higher than that of the 7th AJCC system (8.8% vs 5.8%; Table 1). Better separation of survival curves was found among stages for the m7th AJCC classification (Fig. 2c-d). There was the expected worsening in survival as tumor stage increased (Table 2). In addition, an increase in HRs was observed in the m7th AJCC staging classification by multivariable analyses, although statistically significant difference was not observed between stages I and II (compared with stage I disease; HR for stage II =1.23, 95% CI = 0.73–2.06, *P* = 0.44; HR for stage III =2.20, 95% CI =1.06–4.56, *P* = 0.03; HR for stage IV =4.95, 95% CI =3.20–7.65, *P* < 0.001; Table 3).

Two types of C-indices of different staging systems for pNECs were presented in.

Table 4. One was for local disease only, and the other was for the entire cohort. The respective C- indices using the 7th and m7th staging systems for patients with local disease (stages I–III) were 0.57 (95% CI 0.51–0.63) and 0.59 (95% CI 0.53–0.66), respectively. Also, for the entire cohort, the C- indices based on the 7th staging system (0.626, 95% CI 0.599–0.653), and the m7th staging system (0.627, 95% CI 0.600–0.654) were similar.

**Table 4:** C-indices of Different Staging Systems for pNECs

Staging System	Local, n = 154
7th AJCC	0.58 (0.51–0.63)
m7th AJCC	0.59 (0.53–0.66)
Staging System	All, n = 568
7th AJCC	0.626 (0.599–0.653)
m7th AJCC	0.627 (0.600–0.654)

Values in parentheses are 95% confidence intervals. Abbreviations: *pNECs* poorly differentiated pancreatic neuroendocrine carcinomas; *AJCC* American Joint Committee on Cancer; *m7th AJCC* modified 7th AJCC

## Discussion

Currently, there was no widely acceptable staging system for pNECs, and this was the first consolidation of a data-based process for the revision of the staging classification on pNECs. We tested three TNM staging systems to determine which was superior in terms of performance when a large series of pNECs were used.

Our data demonstrated that the 7th AJCC staging classification has better distribution of pNECs with different stages compared with the ENETS and 8th AJCC staging systems. We observed an increase in HRs by multivariable analyses and decreased median survival time from class I to IV across the 7th AJCC staging system, whereas this trend was not observed for the ENETS and 8th AJCC staging systems. However, the median OS of the patients within stage II varied widely among the different substages in the 7th AJCC system, and patients with stage IIB even had a better prognosis than that of stage IIA. The consistency of the outcomes among the substages became unclear, warranting further modifications and validation. In addition, multivariable cox regression analysis indicated that only the T definitions of the 7th AJCC could allocate patients in four risk groups for the local patients that death risk uniformly progressed from T1 to T4(T1 served as the reference; T2 HR =2.02, 95% CI = 0.58–7.02; T3 HR = 2.27, 95% CI = 0.66–7.84; T4 = 4.16, 95% CI = 1.11–15.52), whereas the trend was not found for the ENETS (T1 served as the reference; T2 HR = 3.04, 95% CI = 0.67–13.89; T3 HR = 4.18, 95% CI = 0.95–18.41; T4 HR = 3.86, 95% CI = 0.88–16.94) and 8th AJCC staging systems(T1 served as the reference; T2 HR = 1.91, 95% CI = 0.72–5.05; T3 = 1.31, 95% CI = 0.48–3.57; T4 = 3.57, 95% CI = 0.89–14.28)(Supplementary Table 2), supporting the adoption of the T definitions of the 7th AJCC in our modified staging system. Moreover, we observed that lymph node status alone was not a significant predictor of survival in univariate and multivariate analysis (Supplementary Table 3; Supplementary Table 4). As with our findings, some studies have already demonstrated the predictive value of lymph nodal status for pNENs was limited and that the nodal stage showed no distinguished differences on the estimated cumulative survival probability [26–29]. However, given that there still existed some patients without lymph node dissection or with insufficient lymph node dissection, lymph node staging was sometimes hard to perform, and deserved to be confirmed in a relatively large sample size.

Therefore, we proposed an adjustment to the 7th AJCC staging classification by maintaining the T and M definitions but adopting a new staging definition system. The proportion of patients with stage I disease using the m7th AJCC staging system was higher than that of the 7th AJCC system (8.8% vs 5.8%). The death rates uniformly progressed from class I to IV, although there was no significant difference between stage I and stage II. Moreover, the discrimination ability for tumor-related death measured by the Harrell C statistics was slightly better for the m7th AJCC staging system relative to the 7th AJCC staging classification. Consequently, these findings suggested that the m7th AJCC staging classification was more suitable for pNECs and easier to use, and thus it should be further investigated.

The creation of new staging system based on modification of the 7th AJCC staging classification will help the stratification for patients with pNECs in the future. However, there remain uncovered situations. Although only the T definition of the 7th AJCC can allocate patients into four risk groups for the local patients without significant differences, the T staging for pNECs was challenged due to impractical and insufficient prognostic correlations for pancreatic ductal adenocarcinoma. Consequently, the definition of T needs to be further optimized according to the biological behavior of pNECs, such as the appropriate cutoff for tumor size. Even so, traditional predictors of outcome being tumor size, extent of tumor and presence of distant metastasis have not been accurate to describe the biology of pNECs. There may be more predictive of survival compared to tumor size, extension of tumor and metastatic status. Up to now, the predictors regarding the pNECs are limited. Besides the tumor stage, multivariate analyses showed that age, race and location of tumor were significantly associated with OS in our study (Supplementary Table 3; Supplementary Table 4). Also, there is no high-quality prognostic risk assessment model for the patients with pNECs. Further efforts are necessary to focus on developing a risk assessment model, which can be offered to clinicians to assess patient prognosis, enhance patient stratification and strengthen the prognosis-based decision making.

We acknowledge several limitations. First, the study was limited by its volume of the data, which may explain the lack of significant differences between stage I and stage II using the m7th AJCC staging system. Second, since this was an opportunistic use of existing data sets, some prognostic factors reported in other studies were not considered to be adjusted for multivariate analysis because of the nonavailability of this information from SEER database, such as Ki67 staining of cancer cells and treatment-related variables. Last, although the SEER database keeps highly accurate records, incorrect coding or erroneous data are also possible. And our study was also limited by its retrospective nature, additional prospective validation will be required to evaluate the modified staging system.

## Conclusions

In conclusion, the present study indicated that the 7th AJCC staging classification was superior in performance relative to the ENETS and 8th AJCC staging systems for pNECs. The 7th AJCC staging system still has room for improvement. A modified staging system is proposed by maintaining the T and M definitions of the 7th AJCC staging system. However, the modified staging system is more accurate and reliable in predicting the prognosis of pNECs. We accept that the study still has some limitations that can only be addressed by the next phase of our work, but, for now, we also believe this new staging system will be a fast and accurate prognostic assessment tool for pNECs to risk-stratify patients and guide treatment.

## Supplementary information

**Additional file 1 Table S1**. The AJCC Staging Definitions, the ENETS Staging Definitions, and the Modified 7th AJCC Staging Definitions Staging Definitions for pNECs **Table S2.** Multivariate Analyses of other predictors of tumor-related death among the local patients (*N* = 154) using the definitions of the ENETS, 8th AJCC, 7th AJCC staging systems for pNECs **Table S3.** Univariate Analyses of other predictors of tumor-related death among 568 patients using the definitions of the ENETS, 8th AJCC, 7th AJCC staging systems for pNECs **Table S4.** Multivariate Analyses of other predictors of tumor-related death among 568 patients using the definitions of the ENETS, 8th AJCC, 7th AJCC staging systems for pNECs.

## Data Availability

The datasets used and/or analysed during the current study are available from the corresponding author on reasonable request.

## Abbreviations

7th: 7th edition
8th: 8th edition
AJCC: American Joint Committee on Cancer
CI: Confidence interval
C-Index: concordance index
CS: Collaborative stage
ENETS: European Neuroendocrine Tumor Society
EOD: Extent of disease
HR: Hazard ratio
M7th: Modified 7th
OS: Overall survival
PNECs: Pancreatic neuroendocrine carcinomas
PNENs: Neuroendocrine neoplasms of the pancreas
PNETs: Pancreatic neuroendocrine tumors
SEER: Surveillance, Epidemiology, and End Results
TNM: Tumor–node–metastasis
