# Supplementary material for: Validation and modification of staging Systems for Poorly Differentiated Pancreatic Neuroendocrine Carcinoma
Source: BMC Cancer. 2020 Mar 6;20:188. doi: 10.1186/s12885-020-6634-9 (PMC7059325; doi:10.1186/s12885-020-6634-9)
Supplement: Supplementary file 1 — Additional file 1 Table S1. The AJCC Staging Definitions, the ENETS Staging Definitions, and the Modified 7th AJCC Staging Definitions Staging Definitions for pNECs Table S2. Multivariate Analyses of other predictors of tumor-related death among the local patients (N = 154) using the definitions of the ENETS, 8th AJCC, 7th AJCC staging systems for pNECs Table S3. Univariate Analyses of other predictors of tumor-related death among 568 patients using the definitions of the ENETS, 8th AJCC, 7th AJCC staging systems for pNECs Table S4. Multivariate Analyses of other predictors of tumor-related death among 568 patients using the definitions of the ENETS, 8th AJCC, 7th AJCC staging systems for pNECs. [file 12885_2020_6634_MOESM1_ESM.docx]

| **Supplementary Table 1**. The AJCC Staging Definitions, the ENETS Staging Definitions, and the Modified 7th AJCC Staging Definitions Staging Definitions for pNECs | | | | | | | |
| --- | --- | --- | --- | --- | --- | --- | --- |
|  | ENETS Staging Classification |  | 7th AJCC Staging System | | |  | 8th AJCC Staging System |
| T1 | \| Limited to the pancreas, <2 cm \| \| --- \| | T1 | Limited to the pancreas, ≤2 cm in greatest dimension | | | T1 | Tumor≤2 cm in the greatest dimension |
| T2 | Limited to the pancreas, 2–4 cm | T2 | Tumor limited to the pancreas, >2 cm in the greatest dimension | | | T2 | Tumor >2 cm and ≤4 cm in the greatest dimension |
| T3 | Limited to the pancreas, >4 cm or invading duodenum or bile duct | T3 | Tumor extends beyond the pancreas, but without involvement of the celiac axis or the superior mesenteric artery | | | T3 | Tumor >4 cm in the greatest dimension |
| T4 | Tumor invades adjacent structures | T4 | Tumor involves the celiac axis or the superior mesenteric artery  (unresectable primary tumor) | | | T4 | Tumor involves the celiac axis, the superior mesenteric artery, and/or common hepatic artery, irrespective of size |
| N0 | No regional lymph node metastasis | N0 | No regional lymph node metastasis | | | N0 | No regional lymph node metastasis |
| N1 | Regional lymph node metastasis | N1 | Regional lymph node metastasis | | | N1 | Metastasis in 1 to 3 regional lymph nodes |
|  |  |  |  | | | N2 | Metastasis in _4 regional lymph nodes |
| M0 | No distant metastasis | M0 | No distant metastasis | | | M0 | No distant metastasis |
| M1 | Distant metastasis | M1 | Distant metastasis | | | M1 | Distant metastasis |
|  | ENETS |  | 7th AJCC | Modified 7th AJCC | |  | 8th AJCC |
| I | T1N0M0 | IA | T1N0M0 | IA | T1NanyM0 | IA | T1N0M0 |
| IIA | T2N0M0 | IB | T2N0M0 | IB | T2NanyM0 | IB | T2N0M0 |
| IIB | T3N0M0 | IIA | T3N0M0 | II | T3NanyM0 | IIA | T3N0M0 |
| IIIA | T4N0M0 | IIB | T1-3N1M0 |  |  | IIB | T1-3N1M0 |
| IIIB | TanyN1M0 | III | T4NanyM0 | III | T4NanyM0 | III | TanyN2M0, T4NanyM0 |
| IV | TanyNanyM1 | IV | TanyNanyM1 | IV | TanyNanyM1 | IV | TanyNanyM1 |
| Abbreviations: AJCC, American Joint Committee on Cancer ; ENETS, European Neuroendocrine Tumor Society ; pNECs , poorly differentiated pancreatic neuroendocrine carcinomas ; M, distant metastasis; N, lymph nodes; T, primary tumor. | | | | | | | |

| **Supplementary Table 2.** Multivariate Analyses of other predictors of tumor-related death among the local patients (N=154) using the definitions of the ENETS, 8th AJCC, 7th AJCC staging systems for pNECs | | | | | | |
| --- | --- | --- | --- | --- | --- | --- |
| Characteristic | ENETS | | 7th AJCC | | 8th AJCC | |
|  | HR (95% CI) | P | HR (95% CI) | P | HR (95% CI) | P |
| Age, years |  |  |  |  |  |  |
| < 60 | 1 |  | 1 |  | 1 |  |
| ≥ 60 | 1.59 (0.98- 2.58) | 0.06 | 1.65 (1.02-2.69) | 0.04 | 1.73 (1.07-2.79) | 0.03 |
| Sex |  |  |  |  |  |  |
| Female |  |  |  |  |  |  |
| Male | 1.07(0.66 -1.74) | 0.77 | 1.03 (0.64-1.66) | 0.91 | 1.08 (0.66-1.76) | 0.76 |
| Race |  |  |  |  |  |  |
| Black | 1 |  | 1 |  |  |  |
| White | 0.89 (0.42-1.88) | 0.76 | 0.98 (0.47-2.07) | 0.96 | 0.91 (0.43-1.91) | 0.80 |
| Other | 0.93 (0.36-2.38) | 0.88 | 1.12 (0.43-2.96) | 0.81 | 1.18 (0.45-3.11) | 0.74 |
| Location |  |  |  |  |  |  |
| Body and tail | 1 |  |  |  |  |  |
| Head | 1.96 (1.09-3.52) | 0.02 | 1.97 (1.12-3.48) | 0.02 | 2.14 (1.20-3.83) | 0.01 |
| Others | 1.37 (0.63-2.99) | 0.43 | 1.38 (0.64-2.98) | 0.41 | 1.22 (0.56-2.63) | 0.62 |
| T |  |  |  |  |  |  |
| T1 | 1 |  | 1 |  | 1 |  |
| T2 | 3.04(0.67-13.89) | 0.15 | 2.02 (0.58-7.02) | 0.27 | 1.91 (0.72-5.05) | 0.20 |
| T3 | 4.18(0.95-18.41) | 0.06 | 2.27 (0.66-7.84) | 0.19 | 1.31 (0.48-3.57) | 0.59 |
| T4 | 3.86(0.88-16.94) | 0.07 | 4.16 (1.11-15.52) | 0.03 | 3.57 (0.89-14.28) | 0.07 |
| N |  |  |  |  |  |  |
| N0 |  |  |  |  |  |  |
| N1 | 0.74 (0.45-1.22) | 0.23 | 0.87 (0.52-1.46) | 0.60 | 0.52 (0.28- 0.97) | 0.04 |
| N2 |  |  |  |  | 1.61 (0.87- 2.98) | 0.13 |
| Abbreviations: AJCC, American Joint Committee on Cancer; ENETS, European Neuroendocrine Tumor Society; pNECs, poorly differentiated pancreatic neuroendocrine carcinomas; HR, hazard ratio; CI, confidence interval; N, lymph nodes; T, primary tumor. | | | | | | |

| **Supplementary Table 3.** Univariate Analyses of other predictors of tumor-related death among 568 patients using the definitions of the ENETS, 8th AJCC, 7th AJCC staging systems for pNECs | | |
| --- | --- | --- |
| Characteristic | HR (95% CI) | p |
| Age, years |  |  |
| < 60 | 1 |  |
| ≥ 60 | 1.63（1.33-1.99） | <0.001 |
| Sex |  |  |
| Female | 1 |  |
| Male | 1.07（0.88-1.30） | 0.52 |
| Race |  |  |
| Black | 1 |  |
| White | 0.77（0.58-1.04） | 0.25 |
| Other | 0.79（0.53-1.18） | 0.08 |
| Location |  |  |
| Body and tail | 1 |  |
| Head | 1.38 (1.09-1.74) | 0.01 |
| Others | 1.76 (1.37-2.27) | <0.001 |
| Tumor size, cm | 1.03 (0.99-1.06) | 0.14 |
| Lymph nodes |  |  |
| Negative | 1 |  |
| Positive | 0.76 (0.61 -0.95) | 0.02 |
| Unknown | 1.90(1.48-2.42) | <0.001 |
| T (ENETS) |  |  |
| T1 | 1 |  |
| T2 | 5.52(1.72-17.70) | 0.004 |
| T3 | 5.24(1.65 -6.68) | 0.01 |
| T4 | 4.44 (1.41-13.95) | 0.01 |
| Unknown | 10.92(3.48-34.29) | <0.001 |
| T (8th AJCC) |  |  |
| T1 | 1 |  |
| T2 | 1.52(0.88-2.63) | 0.13 |
| T3 | 1.36(0.79-2.34) | 0.27 |
| T4 | 2.13(1.13 -4.01) | 0.02 |
| Unknown | 3.53(2.07-5.99) | <0.001 |
| T (7th AJCC) |  |  |
| T1 | 1 |  |
| T2 | 2.27(1.05-4.93) | 0.04 |
| T3 | 1.93(0.90-4.14) | 0.09 |
| T4 | 2.60(1.19-5.70) | 0.02 |
| Unknown | 4.92(2.30-10.51) | <0.001 |
| Metastases |  |  |
| M0 | 1 |  |
| M1 | 3.78(2.91-4.92) | <0.001 |
| Abbreviations: AJCC, American Joint Committee on Cancer; ENETS, European Neuroendocrine Tumor Society; pNECs, poorly differentiated pancreatic neuroendocrine carcinomas; HR, hazard ratio; CI, confidence interval; T, primary tumor. | | |

| **Supplementary Table 4.** Multivariate Analyses of other predictors of tumor-related death among 568 patients using the definitions of the ENETS, 8th AJCC, 7th AJCC staging systems for pNECs | | | | | | |
| --- | --- | --- | --- | --- | --- | --- |
| Characteristic | ENETS | | 7th AJCC | | 8th AJCC | |
|  | HR(95% CI) | P | HR (95% CI) | P | HR (95% CI) | P |
| Age, years |  |  |  |  |  |  |
| < 60 | 1 |  | 1 |  | 1 |  |
| ≥ 60 | 1.59 (1.29- 1.95) | <0.001 | 1.63 (1.33-2.01) | <0.001 | 1.64 (1.34-2.02) | <0.001 |
| Sex |  |  |  |  |  |  |
| Female |  |  |  |  |  |  |
| Male | 1.13(0.93 -1.38) | 0.22 | 1.13 (0.93-1.38) | 0.22 | 1.13 (0.92-1.38) | 0.24 |
| Race |  |  |  |  |  |  |
| Black | 1 |  | 1 |  |  |  |
| White | 0.64 (0.47-0.86) | 0.004 | 0.63 (0.46-0.85) | 0.003 | 0.65 (0.48-0.88) | 0.01 |
| Other | 0.67 (0.44-1.02) | 0.06 | 0.67 (0.44-1.01) | 0.06 | 0.69 (0.46-1.04) | 0.08 |
| Location |  |  |  |  |  |  |
| Body and tail | 1 |  |  |  |  |  |
| Head | 1.52 (1.19-1.94) | <0.001 | 1.54 (1.21-1.96) | <0.001 | 1.52 (1.19-1.93) | <0.001 |
| Others | 1.46 (1.12-1.91) | 0.01 | 1.48 (1.13-1.93) | 0.003 | 1.30 (0.99-1.71) | 0.06 |
| T |  |  |  |  |  |  |
| T1 | 1 |  | 1 |  | 1 |  |
| T2 | 4.25 (1.32-13.73) | 0.02 | 1.66 (0.76-3.63) | 0.21 | 1.53 (0.88-2.66) | 0.14 |
| T3 | 3.86 (1.20-12.41) | 0.02 | 1.69 (0.77 -3.69) | 0.19 | 1.16 (0.67-2.01) | 0.61 |
| T4 | 3.36 (1.05-10.70) | 0.04 | 1.62 (0.73-3.61) | 0.24 | 1.44 (0.75-2.75) | 0.28 |
| Unknown | 4.30 (1.34-13.82) | 0.01 | 1.95 (0.88-4.31) | 0.10 | 1.59 (0.91-2.79) | 0.10 |
| N |  |  |  |  |  |  |
| N0 |  |  |  |  |  |  |
| N1 | 0.85 (0.68-1.07) | 0.17 | 0.84 (0.66-1.06) | 0.14 | 0.53 (0.36-0.78) | 0.001 |
| N2 |  |  |  |  | 0.85 (0.57-1.27) | 0.44 |
| Unknown | 1.21 (0.90-1.61) | 0.20 | 1.20 (0.90-1.60) | 0.21 | 1.21 (0.96 -1.53) | 0.11 |
| M |  |  |  |  |  |  |
| M0 | 1 |  | 1 |  |  |  |
| M1 | 3.40 (2.55- 4.55) | <0.001 | 3.44 (2.56- 4.63) | <0.001 | 2.99 (2.20- 4.06) | <0.001 |
| Abbreviations: AJCC, American Joint Committee on Cancer; ENETS, European Neuroendocrine Tumor Society; pNECs, poorly differentiated pancreatic neuroendocrine carcinomas; HR, hazard ratio; CI, confidence interval. M, distant metastasis; N, lymph nodes; T, primary tumor. | | | | | | |
